# Noncoding RNA profiling in omentum adipose tissue from obese patients and the identification of novel metabolic biomarkers

**DOI:** 10.3389/fgene.2025.1533637

**Published:** 2025-02-06

**Authors:** Yongjiao Zhang, Ao Chen, Sumei Lu, Dong Liu, Xiaolei Xuan, Xiaofei Lei, Mingwei Zhong, Fei Gao

**Affiliations:** ^1^ Department of Clinical Laboratory Medicine, The First Affiliated Hospital of Shandong First Medical University and Shandong Provincial Qianfoshan Hospital, Shandong Medicine and Health Key Laboratory of Laboratory Medicine, Jinan, China; ^2^ School of Medical Laboratory, Shandong Second Medical University, Weifang, Shandong, China; ^3^ Department of Gastroenterology, The First Affiliated Hospital of Shandong First Medical University and Shandong Provincial Qianfoshan Hospital, Jinan, China; ^4^ Department of General Surgery, The First Affiliated Hospital of Shandong First Medical University and Shandong Provincial Qianfoshan Hospital, Jinan, China

**Keywords:** obesity, RNA sequencing (RNA-seq), noncoding RNA (ncRNA), omental adipose tissue, lncRNA

## Abstract

**Background:**

Obesity, a prevalent metabolic disorder, is linked to perturbations in the balance of gene expression regulation. Noncoding RNAs (ncRNAs), including long noncoding RNAs (lncRNAs), circular RNAs (circRNAs), and microRNAs (miRNAs), play pivotal roles in regulating gene expression. The aim of this study was to identify additional ncRNA candidates that are implicated in obesity, elucidating their potential as key regulators of the pathogenesis of obesity.

**Methods:**

We identified distinct ncRNA expression profiles in omental adipose tissue in obese and healthy subjects through comprehensive whole-transcriptome sequencing. Subsequent analyses included functional annotation with GO and KEGG pathway mapping, validation via real-time quantitative polymerase chain reaction (qRT‒PCR), the exploration of protein‒protein interactions (PPIs), and the identification of key regulatory genes through network analysis.

**Results:**

The results indicated that, compared with those in healthy individuals, various lncRNAs, circRNAs, and miRNAs were significantly differentially expressed in obese subjects. Further verifications of top changed gene expressions proved the most genes’ consistence with RNA-sequencing including 11 lncRNAs and 4 circRNAs. Gene network analysis highlighted the most significant features associated with metabolic pathways, specifically ENST00000605862, ENST00000558885, and ENST00000686149. Collectively, our findings suggest potential ncRNA therapeutic targets for obesity, including ENST00000605862, ENST00000558885, and ENST00000686149.

## 1 Introduction

Obesity has become a prevalent chronic disease worldwide that is often accompanied by numerous complications. The World Health Organization (WHO) defines obesity as an excessive accumulation of body fat that may be detrimental to health, with a diagnosis based on a body mass index (BMI) of ≥30 kg/m^2^ (Zou and Pitchumoni, 2023). In 2023, an analysis of the obesity epidemiology in China revealed that 34.8% of the population was overweight and 14.1% was obese. Furthermore, individuals with a high BMI were found to have a higher prevalence of comorbidities, including diabetes, hypertension, dyslipidemia, fatty liver disease, chronic kidney disease, and carotid plaques (Chen et al., 2023). Therefore, effective treatment for obesity remains a critical public health priority.

Obesity is characterized by excess body fat, which is categorized into white adipose tissue (WAT) and brown adipose tissue (BAT) on the basis of its form and function (Squillaro et al., 2020). The process of adipose differentiation, or fat formation, is intricately regulated by the genome and involves both coding and noncoding RNAs (ncRNAs). A majority of the human genome is transcribed into RNAs that do not encode proteins; it is estimated that 51.8% of the human genome can be transcribed, with only up to 1.2% of genes coding for proteins, leaving the remainder as ncRNAs. NcRNAs are classified into various categories on the basis of their length, shape, and location, and the four major types are long noncoding RNA (lncRNA), circular RNA (circRNA), microRNA (miRNA), and PIWI-interacting RNA (piRNA) (Yan and Bu, 2021). LncRNAs, which are longer than 200 nucleotides, play crucial roles in gene regulation through cis- or trans-acting mechanisms (Khan et al., 2016). MiRNAs are short RNA molecules, 19 to 25 nucleotides in length, that modulate the posttranscriptional silencing of target genes and influence the expression of numerous genes within functional pathways (Lu and Rothenberg, 2018). CircRNAs represent a novel class of covalently closed, single-stranded endogenous ncRNAs, whereas piRNAs constitute a broad class of small ncRNAs associated with PIWI clade proteins in the Argonaute family (Wang et al., 2022). Additionally, lncRNAs, circRNAs, miRNAs, and mRNAs can form complex, miRNA-centered regulatory ceRNA networks involved in posttranscriptional regulation. Thus, the complex role of ncRNAs in the pathogenesis of obesity remains largely enigmatic, prompting us to conduct comprehensive ncRNA profiling in omental adipose tissue and to identify novel biomarkers for obesity.

Emerging evidence demonstrates the essential function of non-coding RNAs in regulation of adipose development and adipogenesis. Multiple LncRNAs were found to be regulated by transcription factors such as PPARγ and CEBPα during adipogenesis. For example, LncRNAs SRA(steroid receptor RNA activator),Plnc1 and IMFNCR regulate the transcriptional activity of PPAR and are involved in adipocyte differentiation (Fadini et al., 2010; Zhu et al., 2018; Zhang M. et al., 2019). And LncRNA TINCR enhances adipogenic differentiation in adipose tissue-derived mesenchymal stem cells (ADSC) by adsorbing miR-31 to promote the transcriptional activity of C/EBPα (Liu et al., 2018). While LncRNA CAAlnc1 inhibits adipogenesis in C3H10T1/2 cells by binding to HuR and blocking the transcription of RRARγ and C/EBPα (Shen et al., 2019), lncRNA XIST inhibits high-fat diet-induced obesity by binding to C/EBPα (Wu et al., 2022). Studies on miRNAs in obesity have found that MiRNAs are involved in the regulation of adipogenesis. Among them, MiR-146b is highly expressed in mature adipose and can affect visceral adipogenesis (Chen et al., 2014). MiR-210 can inhibit WNT signaling by targeting Tcf7l2, thus promoting adipogenesis (Qin et al., 2010). MiR-152 has been shown to inhibit preadipocyte proliferation and promote lipid accumulation in 3T3-L1 (Fan et al., 2019). Overexpression of miR-124a results in decreased lipid metabolism and cellular triacylglycerol (TG) accumulation (Das et al., 2015). In addition, studies on circRNAs and obesity have found that circRNAs are also important regulators of adipocyte development and function (Ru et al., 2023). For example, circSAMD4A can promote adipogenesis through miR-138-5p/EZH2 axis (Liu et al., 2020). In addition, deletion of circH19 enhances adipogenic differentiation of human adipose-derived stem cells (ADSC) (Zhu et al., 2020). However, adipogenesis is considered to be a complex process, and the study of ncRNAs is still in the preliminary stage, and many ncRNAs related to adipogenesis and their functions and regulatory mechanisms still need to be further investigated in greater depth.

In this study, we established comprehensive ncRNA profiles from human greater omentum adipose tissue and compared the profiles between obese individuals with healthy controls via whole-transcriptome sequencing. Subsequent validation via qRT‒PCR substantiated the sequencing results, reinforcing the reliability of our findings. These insights not only validate the importance of ncRNAs but also lay a theoretical foundation for further functional exploration of the roles of lncRNAs, circRNAs, and miRNAs in obesity, potentially offering novel perspectives on the pathogenesis of this condition.

## 2 Materials and methods

### 2.1 Ethics statement and sample description

The current study included a clinical cohort of 10 participants, consisting of 5 healthy and 5 obese individuals aged between 27 and 68 years. The body mass index (BMI) was calculated as the weight in kilograms divided by the square of the height in meters (kg/m^2^). Participants with a BMI between 25 and 28 were classified as healthy, whereas those with a BMI exceeding 30 were categorized as obese. Greater omentum adipose tissue samples were obtained from both healthy and obese patients who underwent laparoscopic Chol cystolithotomy in the general surgery department. All participants provided written informed consent, and the study received approval from the Ethics Committee of The First Affiliated Hospital of Shandong First Medical University, with reference number [2022] No. S628.

### 2.2 RNA extraction library construction and sequencing

RNA sequencing was carried out by LC-Bio Technologies Company in Hangzhou, China. Total RNA extraction was performed with TRIzol reagent (Thermo Fisher Scientific, 15596018), followed by ribosomal RNA depletion with the Ribo-Zero Gold rRNA Removal Kit (Illumina, MRZG12324, San Diego, United States). The residual RNA was then fragmented into short segments and subjected to reverse transcription. The final cDNA library had an average insert size of 300 ± 50 bp and was sequenced on the Illumina NovaSeq 6000 platform. To ensure high-quality clean reads, the sequences were refined with Cutadapt (https://cutadapt.readthedocs.io/en/stable/,version:cutadapt-1.9). The raw RNA sequencing data, provided in fastq format by LC-Bio, were analyzed by the authors with DESeq2 software to assess the differences in expression between the two groups.

### 2.3 LncRNA identification

The first step involved filtering out any transcripts that overlapped with known mRNAs, known lncRNAs, and RNAs shorter than 200 bp. We then utilized CPC 0.9-r2 (http://cpc2.cbi.pku.edu.cn) and CNCI 2.0 (https://github.com/www-bioinfo-org/CNCl#install-cnci) with the default parameters to predict novel transcripts with coding potential. Transcripts with CPC scores <0.5 and CNCI scores <0 were retained and classified as novel lncRNAs. Additionally, the remaining transcripts with class codes (i, j, o, u, x) were considered novel lncRNAs.

### 2.4 Different expression analysis of lncRNAs

Differential expression analysis was conducted using DESeq2 software to compare two distinct groups. mRNAs, and lncRNAs were identified as differentially expressed if they met the criteria of a false discovery rate (FDR) less than 0.05 and an absolute fold change of at least 2. Subsequently, the differentially expressed coding RNAs underwent enrichment analysis for GO functions and KEGG pathways to elucidate their biological roles and pathways.

### 2.5 GO enrichment analysis

Gene Ontology (GO) is an internationally recognized and standardized framework for the functional classification of genes. It provides a continuously updated controlled vocabulary and rigorously defined concepts that facilitate the comprehensive characterization of gene attributes and their corresponding products across diverse organisms. The GO framework encompasses three primary ontologies: molecular function (MF), cellular component (CC), and biological process (BP). Each fundamental element of GO is referred to as a GO term, which is assigned to a specific ontology category.

GO enrichment analysis was used to identify the GO terms that exhibited significant enrichment among the differentially expressed genes (DEGs) compared with the genomic background, allowing the isolation of DEGs associated with particular biological functions. Initially, all DEGs were mapped to GO terms in the Gene Ontology database (http://www.geneontology.org/), and the number of genes corresponding to each term was quantified. The identification of significantly enriched GO terms within the DEGs relative to the genomic background was achieved through the application of a hypergeometric test. The formula for calculating the P value is as follows:

$$P=1-\sum_{i=0}^{m-1}\frac{\left(\begin{array}{c} M\\ i \end{array}\right)\left(\begin{array}{c} N-M\\ n-i \end{array}\right)}{\left(\begin{array}{c} N\\ n \end{array}\right)}$$

In this context, N represents the total count of the genes that possess GO annotations, whereas n denotes the number of DEGs within this total. M signifies the aggregate number of genes associated with specific GO terms, and m indicates the quantity of DEGs within this subset. Specifically, N corresponds to the total background gene count (denoted as the TB gene number), n refers to the total number of significant genes (TS gene number), M represents the background gene count (B gene number), and m indicates the number of significant genes (S gene number). GO terms that fulfilled the criterion of p < 0.05 were classified as significantly enriched among the DEGs. This analytical approach successfully identified the primary biological functions represented by these DEGs.

### 2.6 Pathway enrichment analysis (KEGG)

Genes typically engage in interactions that contribute to specific biological functions. Analyzing pathways provides deeper insights into the biological roles of genes. The Kyoto Encyclopedia of Genes and Genomes (KEGG) serves as the principal public database related to biological pathways. Pathway enrichment analysis allows the identification of significantly enriched metabolic and signal transduction pathways among DEGs compared with the entire genomic background. The formula employed for these calculations mirrors that utilized in GO analysis. In this context, N denotes the total number of genes annotated within the KEGG, whereas n represents the count of DEGs within that total. Furthermore, M signifies the total number of genes assigned to specific pathways, and m indicates the number of DEGs within those pathways. Pathways that satisfied the condition of p < 0.05 were classified as significantly enriched pathways within the DEGs.

### 2.7 Localization of noncoding RNAs in the genome (Circos plot)

One diagram was generated to show the localization and abundance of noncoding RNAs in the genome with the program Circos.

### 2.8 Protein‒protein interaction analysis and the identification of hub genes

A protein‒protein interaction (PPI) network of metabolic-associated DEGs was constructed with STRING (http://cn.string-db.org). Cytoscape software was used to visualize the PPI network. The network was ranked on the basis of the degree parameter calculated with the CytoNCA plugin of Cytoscape. The MCODE plugin in Cytoscape detected highly interconnected gene modules. To identify hub genes in the PPI network, the “cytoHubba” plugin of Cytoscape was used to score the genes. Finally, the results of the edge percolated component (EPC), degree, maximum neighborhood component (MNC), maximal clique centrality (MCC), closeness and radiality were used for the hub genes.

### 2.9 Real-time quantitative polymerase chain reaction (qRT‒PCR)

The top 10 upregulated and top 10 downregulated differentially expressed (DE) lncRNAs and the top 5 upregulated and top 5 downregulated DE circRNAs were all assessed as candidates via RT‒qPCR. Total RNA was extracted from human adipose samples via TRIzol^TM^ reagent. First-strand complementary DNA was synthesized with the use of a PrimeScript^TM^ RT reagent kit. Afterward, qPCR was carried out with SuperReal PerMix Plus. To calculate the relative expression of the candidate genes, a twofold correlation method was used with β-actin as the reference gene. The qPCR primers used are listed in Table 1.

**TABLE 1:** PCR primers for RNA sequencing verification.

ncRNA name	Forward	Reverse
ENST00000650128	CAG​CGT​GAT​AGC​CTG​ACC​TT	TGC​CAT​CGG​AAA​CCA​GGA​G
ENST00000666290	GAC​TGC​TAC​ACC​AGG​CAC​TC	TCT​GTC​TCG​TTT​CTG​TCC​GC
ENST00000688903	GAC​AGA​AGC​CAT​TGA​GAG​AGC​A	AAACACCGACAATTAAGATGGAGTG AAACACCGACAATTAAGATGGAGTG
ENST00000556989	TTT​AGG​TGA​CCA​AGG​GTC​CTG	CCA​CAA​GTA​GCG​TCC​ACA​AG
ENST00000683613	GAG​ACA​CAT​CAG​CTT​TGC​ATC​A	ACC​CAA​GTC​AGG​TGA​GAC​AG
ENST00000677331	CCT​CAG​CAC​TGT​CAA​GAC​ATA​AA	ATG​ACC​TTA​AGG​ATC​TGT​GCC​CT
ENST00000642268	CAA​CCT​TTG​GAG​GGG​CTG​T	CAG​CAT​AGC​ACG​CCC​AGA​TA
ENST00000565534	GCTTCGGACCCACACAAC	GAG​GCG​TCA​AAG​GTT​CGT​GA
ENST00000525315	GCC​ACC​CCA​GAG​AGT​TAT​GT	GAC​TCT​CCA​GTG​TTG​CTC​CC
ENST00000558885	TGT​AGC​TGG​AAT​TGT​GTA​AGA​AAG	GAT​TCT​GGG​AGA​TTT​CAT​AGT​CAC
ENST00000635626	ACA​GCC​TAC​TGT​TTG​CAT​GG	AAC​CTC​TTA​CAA​CCT​ACC​GCT
ENST00000621909	CTG​GAA​GCT​GGG​AAA​TGA​TAT​GT	TCC​CAG​CTA​TGA​TGC​CTA​TTT​CC
ENST00000605862	GAT​GAA​AGG​GAG​AGT​ATT​CAC​ACA	TCC​TGT​CAT​TCC​ACC​CAA​GG
ENST00000663248	CTG​CCA​CCA​CGG​GTG​AAA​GA	TCT​TGA​AAG​GAG​GAG​AAG​CCT​G
ENST00000686149	AAA​TGG​TAA​ACA​CTG​CCC​TGG	GTA​GGT​GAC​ATT​AGC​CCT​GTT​C
ENST00000446355	TGG​AGG​GAG​GGA​ACC​TTG​TG	TCC​AGA​ATG​CCC​CTG​ACC​TTA
ENST00000483415	GCA​GCA​GAG​TTC​ATT​CCG​ACA	CTC​TTA​CCC​CAT​TCC​ACC​GC
ENST00000554988	GCGGGAACACCCAGCG	CTG​ATG​AGC​TTC​CCT​CCG​C
ENST00000544278	TCT​GAT​GAC​CCG​GCT​TCG​T	AGT​AGC​AGC​CCC​ATA​GAA​AGC
ENST00000487106	CAG​ACG​TTT​TAC​TCC​CCG​CT	TCC​CTC​GTT​ATT​CAG​CAG​CC
hsa_circ_0000722	GGAGGAGCCCAGAGGCAT	CTGGTGGCGGGGCTG
hsa_circ_0003245	GTC​ATA​TTC​CAG​GTG​CAA​ATC​ATA	AGG​ACA​CGC​TAC​GAA​GAA​CC
hsa_circ_0021712	GCT​GTT​ACC​CTT​AAA​GTT​GAA​GAA	ATC​TGT​GGT​TCT​GGT​GAG​GG
hsa_circ_0138872	AAG​CAC​AAA​TCT​GGC​ACT​TGA	CTC​CAC​TTG​TCA​AGC​CTG​C
hsa_circ_0011680	TGG​CAG​ATG​TAA​GAG​AAT​AGA​CAT	CGA​CGT​TCA​ATA​TCA​AGG​CGG
hsa_circ_0003268	CAC​AAA​TAC​GTA​CAG​GTT​TGC​C	TGG​CAA​TGG​AGA​GTG​ACA​GG
hsa_circ_0004281	TCC​ACG​AGA​TGG​AAA​CAT​TGC	ATC​TGT​CGG​AGT​CCC​TCC​AG
hsa_circ_0011708	GGC​CTG​GGA​CCG​AAT​TTC​AA	GTT​TGC​CAA​GTC​TCC​CCA​CT
hsa_circ_0003410	GGA​TGA​ATG​CAT​AGT​GGC​CC	TCT​GTG​GTT​TTT​CCC​GAG​CA
hsa_circ_0013768	GTT​GGA​TAG​GGG​AGG​GTG​AG	CGG​CCT​CCA​GAT​CCA​CAT​TG

### 2.10 Statistical analysis

Categorical variables are expressed as counts with the corresponding percentages, whereas continuous variables are described as the means and standard deviations (SDs). The comparisons of candidate lncRNAs and circRNAs between groups were conducted with t tests. Pearson correlation was employed in this study. A P value less than 0.05 was considered to indicate statistical significance.

## 3 Results

### 3.1 Clinical data of the participants

On the basis of the sequencing quality data, the study ultimately included 3 obese patients and 4 healthy individuals, with 2 obese patients and 1 healthy patient identified as outliers. The mean BMI significantly differed between the two groups, with an average of 50.43 kg/m^2^ in the obese group and an average of 26.13 kg/m^2^ in the healthy group. The levels of several biochemical indicators, including glucose, glycosylated hemoglobin, triglycerides, total cholesterol, high-density lipoprotein (HDL), and low-density lipoprotein (LDL), were markedly elevated in obese patients compared with the levels in healthy patients (Table 2).

**TABLE 2:** Clinical characteristics of obesity patients and healthy crowd.

Characteristics	Obesity patients	Healthy crowd	*P*-value
BMI(kg/m^2^)	51.557 ± 4.331	25.675 ± 1.362	0.0001
Glucose (mmol/L)	8.133 ± 0.918	4.240 ± 0.449	0.0007
Glycosylated hemoglobin (mmol/L)	7.500 ± 0.265	5.725 ± 0.556	0.0040
Triglyceride (mmol/L)	2.220 ± 0.070	1.158 ± 0.423	0.0084
Total cholesterol (mmoI/L)	7.173 ± 0.700	3.290 ± 0.826	0.0013
HDL (mmol/L)	1.427 ± 0.397	0.770 ± 0.080	0.0210
LDL (mmol/L)	4.753 ± 1.040	2.195 ± 0.803	0.0184

### 3.2 Obesity-related noncoding RNAs in adipose tissue from obese and normal-weight individuals

**FIGURE 1:**
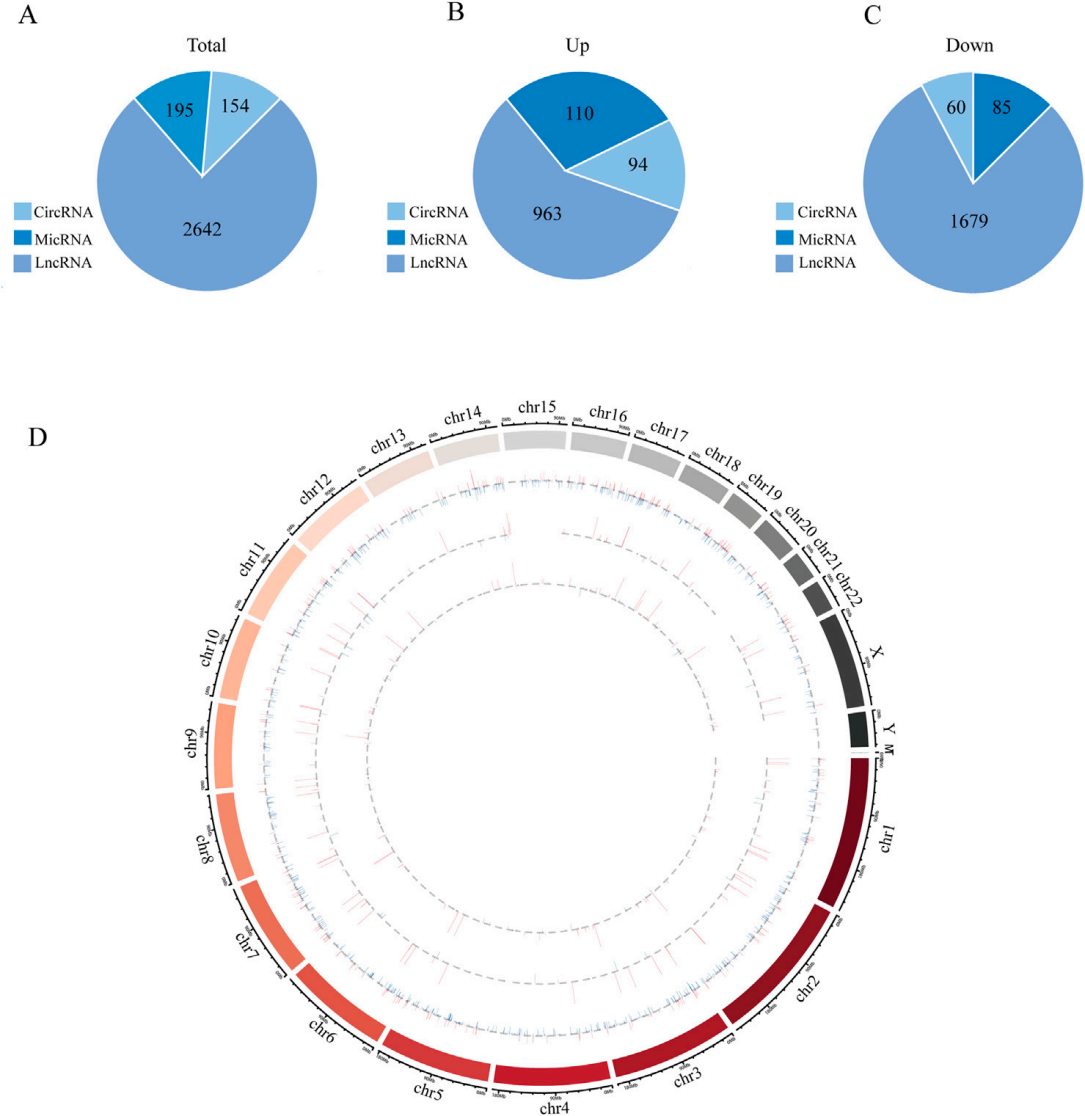
The high-throughput sequencing result between Obesity and Normal group. **(A)** The general expression profiles of lncRNAs, micRNAs and circRNAs between Obesity and Normal group. **(B)** Differentially upregulated lncRNAs, miRNAs and circRNAs between Obesity and Normal group. **(C)** Differentially downregulated lncRNAs, miRNAs and circRNAs between Obesity and Normal group. **(D)** The relative location information of ncRNAs in chromosome, the outermost circle represents chromosome location and then lncRNAs, circRNAs and micRNAs from outside to inside, respectively. The expression profiles of upregulated and downregulated RNAs are marked red and blue, respectively, and the height od each line represents differential expression profiles [log (FDR)].

**FIGURE 2:**
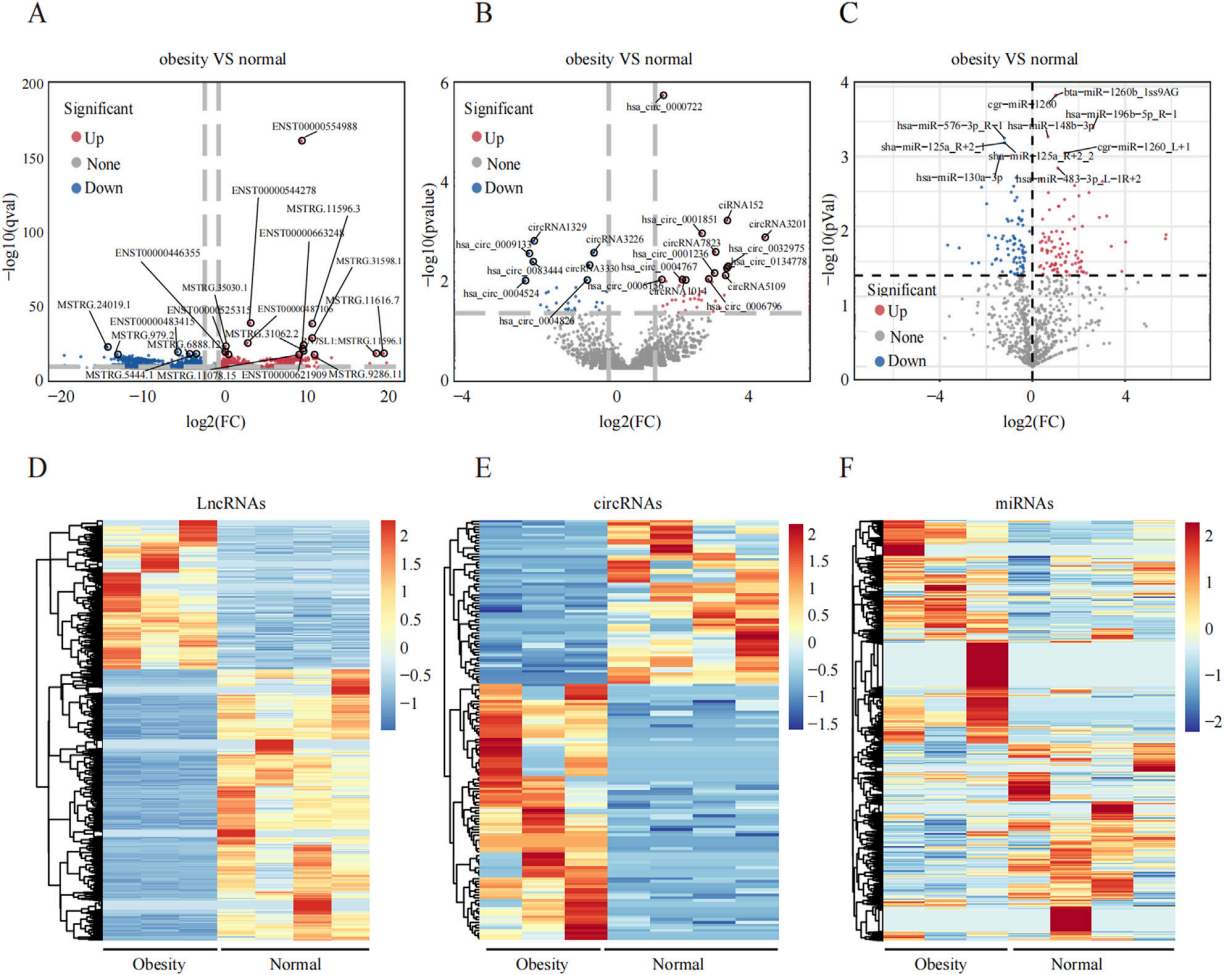
Differential expression profiles of ncRNAs in the greater omentum adipose tissue between obesity and normal individuals. **(A–C)** Volcano plot of DElncRNAs, DEmiRNAs and DEcircRNAs, respectively, the upregulated and downregulated DEncRNAs and marked red and blue. **(D–F)** The hierarchical clustering of DElncRNAs, DEmiRNAs and DEcircRNAs.

An overview of the general ncRNA profiles was initially compiled from the RNA sequencing data, including lncRNAs, miRNAs, and circRNAs. Figure 1A shows that a significant number of ncRNAs were DE (with a fold change ≥2 and P value <0.05), totaling 2642 lncRNAs, 154 circRNAs, and 195 miRNAs. Among these, 963 lncRNAs, 94 circRNAs, and 110 miRNAs were upregulated (Figure 1B), whereas 1679 lncRNAs, 60 circRNAs, and 85 miRNAs were downregulated (Figure 1C). The chromosomal locations of the DE genes are depicted in Figure 1D. Tables 3–5 present the top 10 upregulated and top 10 downregulated ncRNAs, including lncRNAs, circRNAs, and miRNAs, respectively. A graphical outline of the expression characteristics of the lncRNA, cricRNA, micRNA are shown in a hierarchical clustering analysis heatmap and a volcano plot (Figures 2A–F).

**TABLE 3:** The Top 10 upregulated and top 10 downregulated differenciated lncRNAs.

LncRNA segname	Gene	Log2FC	*P*-value	LncRNA segname	Gene	Log2FC	*P*-value
Upregulated				Downregulated			
ENCT00000554988	ENST00000259001	33.72	2.29E-198	ENCT00000483415	EWSR1	−21.29	7.14E-18
ENST00000544278	SNRNP70	25.26	7.88E-44	ENST00000688903	SMAD4	−21.23	2.68E-15
ENST00000487106	LAMTOR2	24.76	4.69E-26	ENST00000686149	KRIT1	−19.18	1.30E-14
ENST00000663248	PAX8-AS1	23.67	3.51E-24	ENST00000605862	ENST00000242588	−18.90	1.92E-14
ENST00000621909	ENST00000277067	22.84	8.70E-19	ENST00000642268	SLCI9A3	−18.02	2.28E-14
ENST00000446355	ENST00000237813	19.02	4.80E-18	ENST00000677331	BRD2	−17.01	7.95E-14
ENST00000525315	CTSB	17.30	1.45E-15	ENST00000683613	ANO5	−16.84	4.27E-13
ENST00000666290	STARD7-AS1	15.47	4.74E-15	ENST00000556989	NUMB	−16.00	6.79E-13
ENST00000635626	LINC01359	15.30	8.08E-15	ENST00000565534	MAN2C1	−15.60	1.18E-12
ENST00000650128	LINC00632	15.03	7.67E-14	ENST00000558885	LINC02157	−15.53	1.27E-12

**TABLE 4:** The Top 10 upregulated and top 10 downregulated differenciated circRNAs.

CircRNA segname	Gene name	Log2FC	*P*-value	CircRNA segname	Gene name	Log2FC	*P*-value
Upregulated				Downregulated			
has-circ-0000722	GSE1	−inf	3.55E-07	has-circ-0138872	rcRNA	−inf	2.29E-05
CircRNA11708		−inf	7.20E-06	CircRNA3410	GARS1-DT	−inf	3.13E-05
CircRNA13768		−inf	2.60E-05	CircRNA3245	CSNK1E	−inf	4.88E-05
has-circ-0004281	FRMD4A	−inf	9.24E-05	circRNA3268	TUT7	−inf	5.17E-04
circRNA11680		−inf	1.48E-04	His-circ-0021712	PDHX	−inf	5.92E-04
circRNA11714		−inf	2.32E-04	circRNA1329	ZC3H12B	inf	9.86E-04
circRNA11606	PDHX	−inf	2.52E-04	has-circ-0001997	PCNX1	inf	1.59E-03
ciRNA152	RAB10	−inf	3.27E-04	circRNA3226	KTFAP3	inf	1.88E-03
has-circ-0137606	NIPSNAP3A	−inf	5.59E-04	circRNA3287	SCAF8	inf	1.90E-03
has-circ-001851	UBAP2	−inf	6.56E-04	has-circ-0009133	MFSD8	inf	1.95E-03

**TABLE 5:** The Top 10 upregulated and top 10 downregulated differenciated micRNAs.

MicRNA segname	Log2FC	*P*-value	MicRNA segname	Log2FC	*P*-value
Up-reguated			Downregulated		
bta-miR-1260b_lss9AG	−inf	1.34E-04	has-miR-576-3p-R_1	−inf	5.47E-04
cgr-miR-1260	−inf	1.34E-04	sha-miR-125R_R+2_2	−inf	6.37E-04
has-miR-196b-5p_R-1	−inf	3.89E-04	sha-miR-125a_R+2_1	−inf	6.37E-04
has-miR-148b-3p	−inf	5.26E-04	hsa-miR-130a-3p	−inf	9.94E-04
cgr-miR-1260_L+1	−inf	8.96E-04	hsa-miR-339-3p	−inf	2.00E-03
has-miR-483-3p_L-1R+2	−inf	1.46E-03	hsa-miR-625-3p	−inf	2.67E-03
hsa-miR-23a-3p_R+1	−inf	2.12E-03	hsa-mir-4485-p3_1ss1CT	−inf	2.75E-03
Bta-miR-2478_lss2TG	−inf	2.27E-03	has-miR-1296-5p	−inf	3.24E-03
mmu-mir-3535-p3	−inf	2.61E-03	hsa-miR-29a-3p_R-1	−inf	3.85E-03
has-miR-194-5p_R+1	−inf	3.28E-03	hsa-miR-342-3p	−inf	4.28E-03

### 3.3 GO and KEGG enrichment analyses of DE noncoding RNAs

**FIGURE 3:**
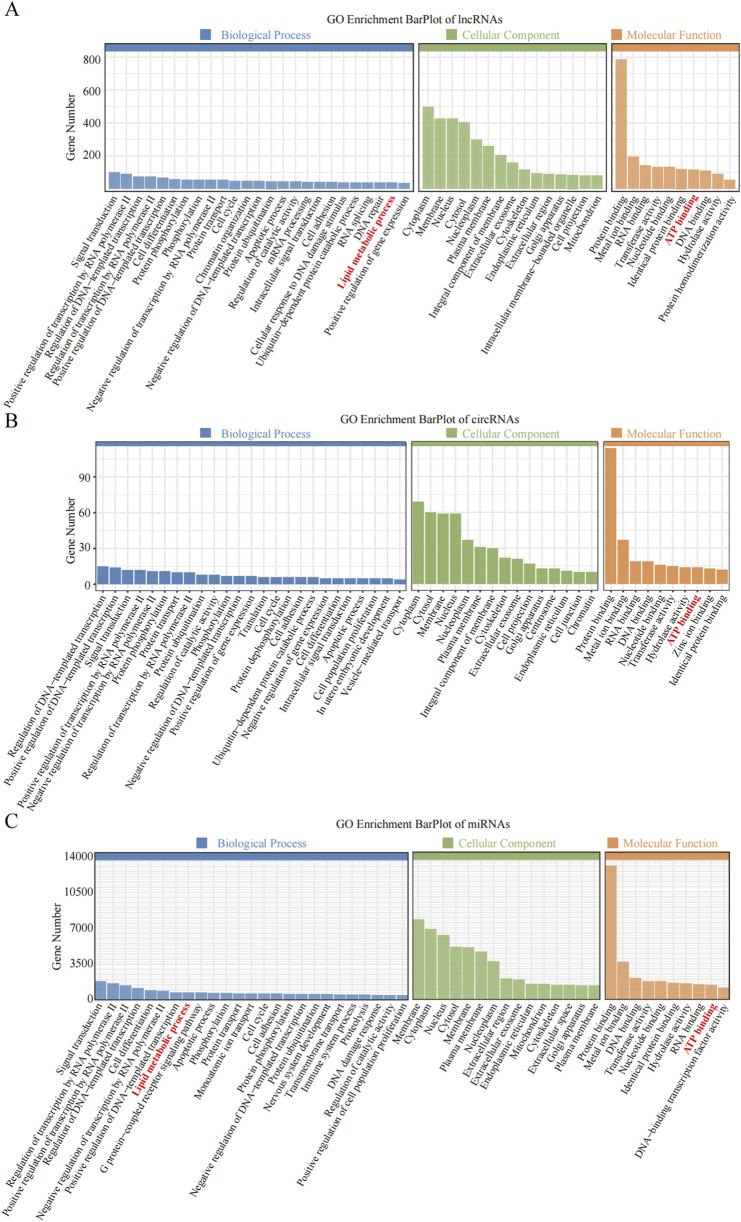
GO enrichment analysis of the differential non-coding RNA. **(A–C)** The *Y*-axis represents the number of DE non-coding RNA (**(A)** lncRNA; **(B)** circRNA; **(C)** miRNA), with individual GO term plotted on the *X*-axis. All GO term were grouped into three categories: biological process, cellular component, and molecular functions.

Enrichment analysis was conducted to uncover the potential roles of the DE lncRNAs, circRNAs, and miRNAs in metabolic pathways. GO analysis of the BP terms revealed that the DE lncRNAs and circRNAs were predominantly enriched in lipid metabolic processes. The GO analysis for cellular components (CCs) indicated that these DE lncRNAs and circRNAs might be associated with cellular compartments such as the “Cytoplasm, Membrane, and Nucleus,” whereas the DE miRNAs were enriched primarily in the Cytoplasmic Cytosol and Membrane. The GO analysis for molecular functions (MFs) suggested that the DE lncRNAs, circRNAs, and miRNAs could be involved in ATP binding (Figures 3A–C; Tables 6–8).

**TABLE 6:** GO enrichment results of differentially expressed genes (lncRNA).

GO	Term	Gene number	*P*-value	Classification
GO:0005829	Cytosol transcription regulatory region sequence-specific DNA binding	403	6.35E-12	CC
GO:0005737	Cytoplasm complex	497	1.24E-11	CC
GO:0005634	Nucleus	425	1.99E-09	CC
GO:0005634	Nucleoplasm death	298	3.70E-09	CC
GO:0003723	RNA binding muscle cell apoptotic process	141	6.29E-09	MF
GO:0006325	Chromatin organization transcription from RNA polymerase II promoter involved in cellular response to chemical stimulus	47	8.53E-09	BP
GO:0005515	Protein binding development	787	6.81E-08	MF
GO:0016607	Nuclear speck multienzyme complex	50	5.40E-07	CC
GO:00043087	Regulation of GTPase activity	19	1.41E-06	BP
GO:0010595	Positive regulation of endothelial cell migtation process	16	1.88E-06	BP

Notes: MF: Molecular Function; BP: biological process; CC: cellular component.

**TABLE 7:** GO enrichment results of differentially expressed genes (circRNA).

GO	Term	Gene number	*P*-value	Classification
GO:0000977	RNA polymerase II transcription regulatory region sequence-specific DNA binding	9	6.21E-04	MF
GO:0030289	Protein phosphatase 4 complex	2	1.85E-03	CC
GO:0010941	Regulation of cell death	2	1.85E-03	BP
GO:1901216	Positive regulation of neuron death	3	2.89E-03	BP
GO:001066	Positive regulation of cardiac muscle cell apoptotic process	2	5.96E-03	BP
GO:1901522	Positive regulation of transcription from RNA polymerase II promoter involved in cellular response to chemical stimulus	2	5.96E-03	BP
GO:0003161	Cardiac conduction system development	2	5.96E-03	BP
GO:0017101	Aminoacyl-tRNA synthetase multienzyme complex	2	5.96E-03	CC
GO:0005813	Centrosome	13	6.03E-03	CC
GO:0042177	Negative regulation of protein catabolic process	3	6.83E-03	BP

Notes: MF: Molecular Function; BP: biological process; CC: cellular component.

**TABLE 8:** GO enrichment results of differentially expressed genes (micRNA).

GO	Term	Number	*P*-value	Classification
GO:0005515	Protein binding	13070	0	MF
GO:0005737	Cytoplasm	6867	0	CC
GO:0005634	Nucleus	6314	0	CC
GO:0005829	Cytosol	5167	0	CC
GO:0005654	Nucleoplasm	3760	9.48E-228	CC
GO:0046872	Metal ion binding	3690	1.87E-225	MF
GO:00016740	Transferase activity	1824	6.02E-125	MF
GO:0016020	Membrance	7799	1.08E-121	CC
GO:0005856	Cytoskeleton	1428	6.88E-116	CC
GO:0006357	Regulation of transcription by RNA polymerase II	1629	1.22E-110	BP

Notes: MF: Molecular Function; BP: biological process; CC: cellular component.

**FIGURE 4:**
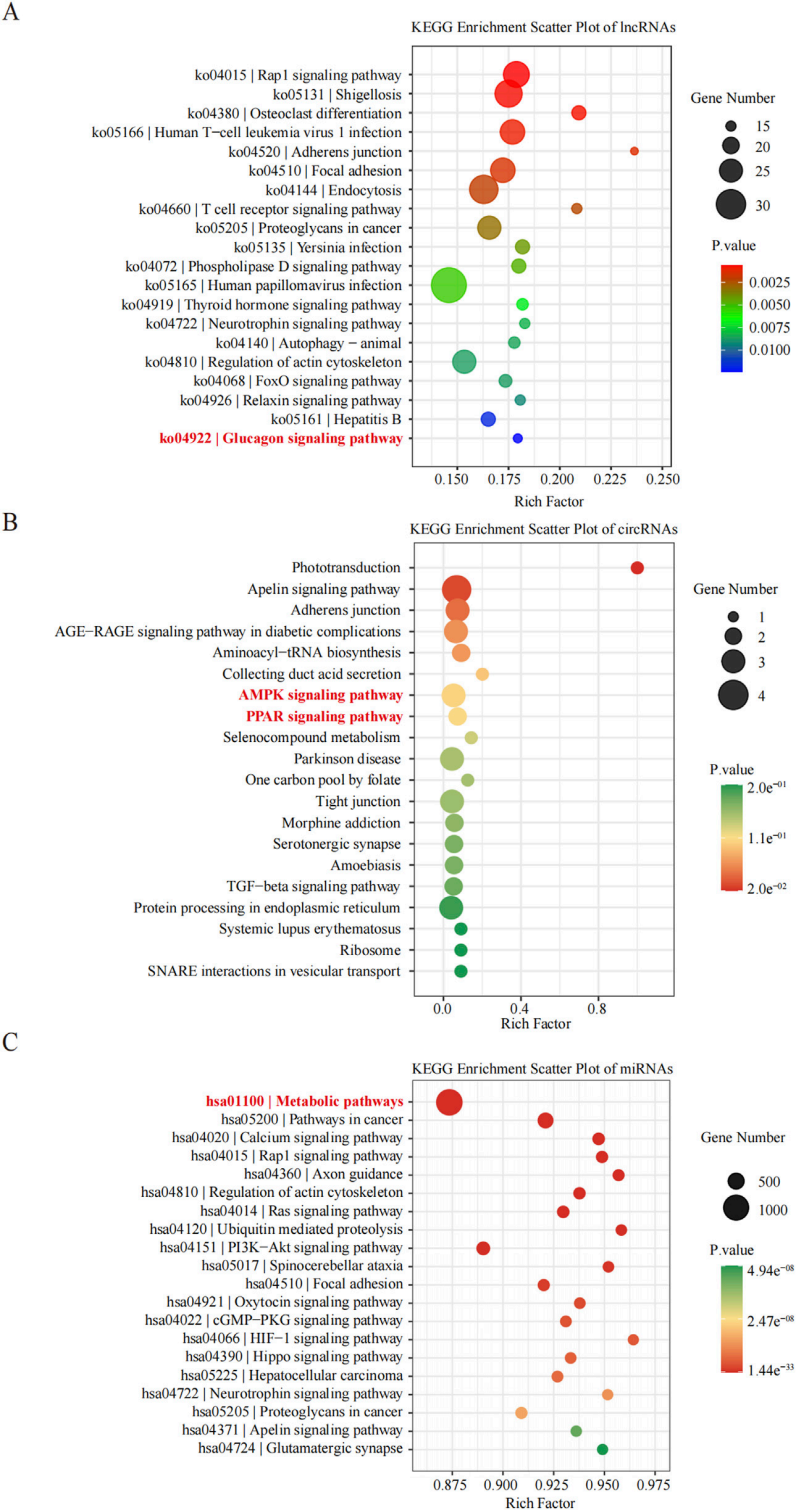
Histogram of Kyoto Encyclopedia of Genes and Genomes (KEGG) pathways enrichment in DE ncRNAs. **(A–C)** The *X*-axis represents the number of DE non-coding RNAs (**(A)** lncRNA; **(B)** circRNA; **(C)** miRNA) annotated in a pathway, with individual KEGG terms shown on the *Y*-axis.

KEGG pathway analyses revealed that the DE lncRNAs were predominantly involved in the “glucagon signaling pathway within metabolic processes” (Figure 4A). The DE circRNAs were chiefly associated with the “AMPK signaling pathway and PPAR signaling pathway” (Figure 4B). AMPK, recognized as a master regulator of energy homeostasis and a principal sensor of nutrient availability, plays a crucial role in promoting catabolic processes while inhibiting anabolic metabolism (Jiang et al., 2021). The PPAR signaling pathway, which is crucial for hepatic lipid metabolism, has been implicated in the dysregulation of hepatic lipid metabolism and multiple metabolic disorders (Mao et al., 2021). The DE miRNAs were involved in metabolic pathways (Figure 4C) (Tables 9–11).

**TABLE 9:** KEGG enrichment pathway results of differentially expressed gene (lncRNA).

Term	ID	GeneRation	*P*-value
Rab1 signaling pathway	ko04015	27/445	6.66E-04
Shigellosis	ko05131	28/445	7.59E-04
Osteoclast differentiation	ko04380	18/445	8.12E-04
Human T-cell leukemia virus 1 infection	ko05166	26/445	9.89E-04
Adherens junction	ko04520	13/445	1.33E-03
Focal adhesion	ko04510	26/445	1.49E-03
Endocytosis	ko04144	29/445	1.97E-03
T cell receptor signaling pathway	ko04660	15/445	2.28E-03
Proteoglycans in cancer	ko05205	25/445	3.17E-03
*Yersinia* infection	ko05135	18/445	4.26E-03
Phospholipase D signaling pathway	ko04072	18/445	4.76E-03
Human papillomavirus infection	ko05165	34/445	5.41E-03
Thyroid hormone signaling pathway	ko04919	16/445	6.86E-03
Neurotrophin signaling pathway	ko04722	15/445	8.23E-03
Autophagy-animal	ko04140	16/445	8.54E-03
Regulation of actin cytoskeleton	ko04810	25/445	8.69E-03
Foxo signaling pathway	ko04068	17/445	8.74E-03
Relaxin signaling pathway	ko04926	15/445	9.21E-03
Hepatitis B	ko05161	18/445	1.17E-02
Glucagon signaling pathway	ko04922	14/445	1.24E-02

**TABLE 10:** KEGG enrichment pathway results of differentially expressed gene (circRNA).

Term	ID	GeneRation	*P*-value
Phototransduction	hsa00190	1/46	2.00E-02
Apelin signaling pathway	hsa00230	4/46	3.01E-02
Adherens junction	hsa00270	3/46	4.69E-02
AGE-RAGE signaling pathway in diabetic complications	hsa00310	3/46	6.58E-02
Aminoacy1-tRNA biosynthesis	hsa00450	2/46	7.01E-02
Collecting duct acid secretion	hsa00620	1/46	9.61E-02
AMPK signaling pathway	hsa00670	3/46	1.04E-01
PPAR signaling pathway	hsa00970	2/46	1.06E-01
Selenocompound metabolism	hsa01100	1/46	1.32E-01
Parkinson disease	hsa01230	3/46	1.48E-01
One carbon pool by folate	hsa01524	1/46	1.49E-01
Tight junction	hsa03010	3/46	1.53E-01
Morphine addiction	hsa03013	2/46	1.61E-01
Serotonergic synapse	hsa03015	2/46	1.68E-01
Amoebiasis	hsa03018	2/46	1.68E-01
TGF-beta signaling pathway	hsa03320	2/46	1.75E-01
Protein processing in endoplasmic reticulum	hsa04010	3/46	1.93E-01
Systemic lupus erythematosus	hsa04014	1/46	2.00E-01
Ribosome	hsa04020	1/46	2.00E-01
SNARE interactions in vesicular transport	hsa04022	1/46	2.00E-01

**TABLE 11:** KEGG enrichment pathway results of differentially expressed gene (micRNA).

Term	ID	GeneRation	*P*-value
Metabolism pathways	hsa01100	1422/7233	1.44E-33
Pathway in cancer	hsa05200	513/7233	1.75E-23
Calcium signaling pathway	hsa04020	233/7233	4.04E-15
Rap1 signaling pathway	hsa04015	204/7233	1.25E-13
Axon guidance	hsa04360	178/7233	3.82E-13
Regulation of actin cytoskeleton	hsa04810	211/7233	1.97E-12
Ras signaling pathway	hsa04014	225/7233	4.95E-12
Ubiquitin mediated proteolysis	hsa04120	138/7233	1.36E-10
P13K-Akt signaling pathway	hsa04151	333/7233	1.92E-10
Spinocerebellar ataxia	hsa05017	139/7233	5.50E-10
Focal adhesion	hsa04510	196/7233	1.54E-09
Oxytocin signing pathway	hsa04921	151/7233	3.01E-09
cGMP-PKG signaling pathway	hsa04022	162/7233	3.96E-09
HIF-1 signaling pathway	hsa04066	108/7233	4.59E-09
Hippo signaling pathway	hsa04390	154/7233	5.87E-09
Hepatocellular carcinoma	hsa05225	165/7233	7.20E-09
Neurotrophin signaling pathway	hsa04722	118/7233	1.30E-08
Proteoglycans in cancer	hsa05205	200/7233	1.60E-08
Apelin signaling pathway	hsa04371	132/7233	4.35E-08
Glutamatergic synapse	hsa04724	112/7233	4.94E-08

### 3.4 Validation of obesity-related ncRNAs in greater omentum adipose tissue

**FIGURE 5:**
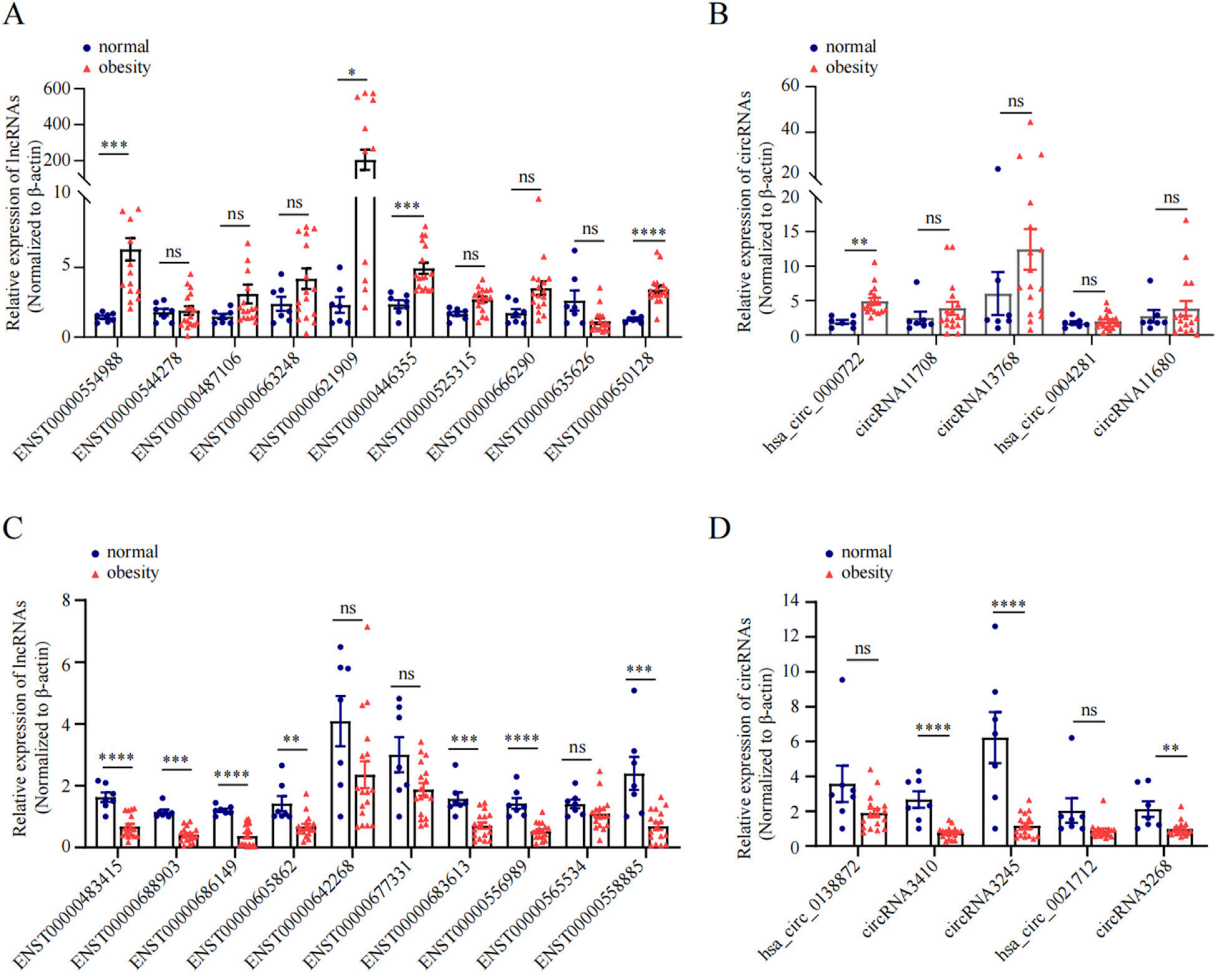
qRT-PCR validation of DE ncRNAs in obese and normal visceral adipose tissues. **(A, C)**: The relative expression of top 10 upregulated and top 10 downregulated lncRNA between obesity and normal body weight individuals. **(B, D)**: The relative expression of top 5 upregulated and top 5 downregulated circRNAs between obesity and normal body weight individuals.

We subsequently conducted qRT-PCR on human omentum adipose tissue samples to validate the top 10 DE lncRNAs and the top 5 DE circRNAs using a criteria by |log_2_FC| ≥2 with adjust *P* < 0.05, including both upregulated and downregulated candidates. As shown in Figure 5, the expression levels of the lncRNAs ENST00000554988, ENST00000446355, and ENST00000650128 and the circRNA hsa_circ_0000722 were significantly greater in obese individuals than in normal-weight individuals (*P* < 0.05), which aligns with the RNA-seq findings. Additionally, the expression of the lncRNAs ENST00000483415, ENST00000688903, ENST00000686149, ENST00000605862, ENST00000642268, ENST00000683613, ENST00000556989, and ENST00000558885 and the expression of the circRNAs circRNA3410, circRNA3245, and circRNA3268 were significantly downregulated in adipose tissue in obese individuals (*P* < 0.05), further corroborating the RNA-seq data.

### 3.5 Bioinformatics analysis of enriched obesity-associated metabolic pathways

**FIGURE 6:**
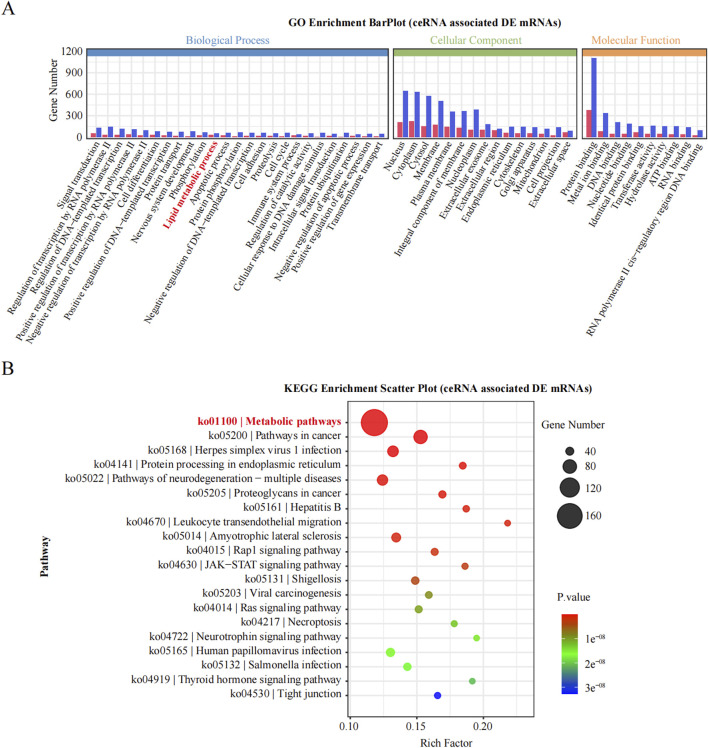
GO and KEGG enrichment analysis of DE genes. **(A)**: The significantly enriched GO of BP, MF and CC. **(B)**: The significantly enriched KEGG pathways of DE gene.

To deepen our understanding of the identified ncRNAs, we constructed a ceRNA regulatory network incorporating the 15 most DE ncRNAs, comprising 11 lncRNAs and 4 circRNAs. We then targeted all the DE mRNAs obtained from RNA-seq to identify those associated with these 15 ceRNAs. Subsequent enrichment analyses were performed to annotate the mRNAs within this ceRNA regulatory network. Notably, a significant number of mRNAs were enriched in lipid metabolic processes, as indicated by the BP term in the GO analysis (Figure 6A). Furthermore, KEGG analysis revealed that the most significantly enriched pathway was metabolic pathways, involving 166 DE mRNAs (Figure 6B). Thus, by targeting DE mRNAs and conducting enrichment analyses, we revealed a close relationship between these 15 ceRNAs and metabolic pathways, suggesting their potential regulatory role in obesity.

### 3.6 PPI network of metabolic pathway-associated DE genes and the identification of hub genes

**FIGURE 7:**
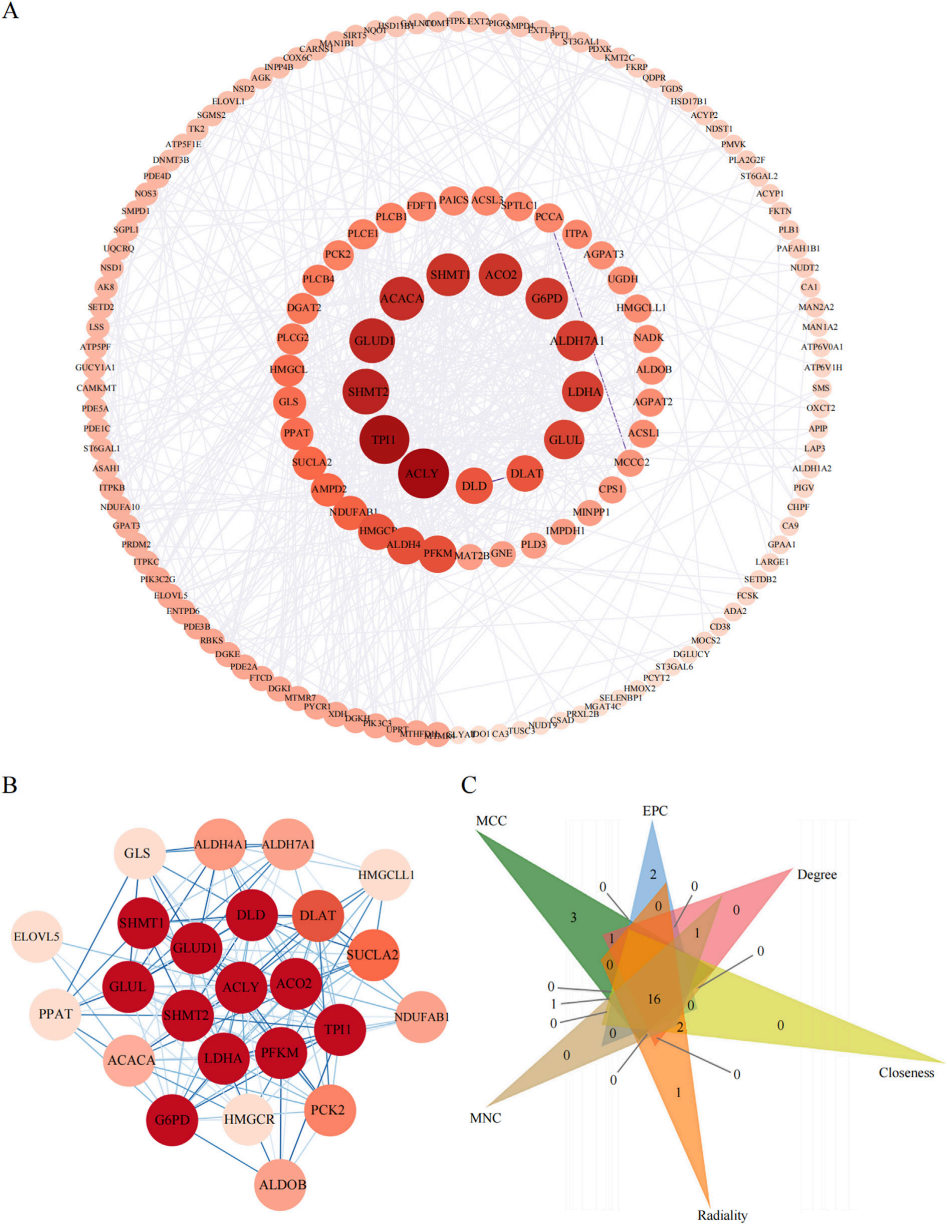
The PPI analyses of metabolic pathway associate EDGs. **(A)**: PPI network of metabolic pathway associate DEGs. **(B)**: The most significant gene clustering module identified by MCODE plugin of Cytoscape software. **(C)**: Venn diagram of 6 similar algorithms (MCC, EPC, Degree, Closeness, Radiality, MNC) in cytoHubba plugin which screened out 16 overlapping hub genes.

To pinpoint the hub genes among the 166 DE genes related to metabolic pathways, we utilized Cytoscape software to construct a PPI network comprising 158 nodes and 624 edges (Figure 7A). We ranked the network on the basis of the degree parameter, which was calculated with the CytoNCA plugin in Cytoscape. Genes with higher degree values were considered to play more significant roles in the network.

We then assessed the coexpression network involving lncRNAs and their target genes. Within this coexpression network, the top three lncRNAs identified were ENST00000605862, ENST00000558885, and ENST00000686149. These findings suggest that these three lncRNAs are closely associated with metabolic pathways, indicating potential functional relatedness or regulatory interactions. The MCODE plugin in Cytoscape was employed to identify the most densely connected gene module, which included 24 common genes (Figure 7B). We subsequently integrated the results from six similar algorithms in cytoHubba to determine the most critical genes within the network. These algorithms included the maximum connectivity (MCC), edge percolated component (EPC), degree, closeness, radiality, and maximum neighborhood component (MNC). The top 25 hub genes identified by these six algorithms were intersected, yielding a final set of 16 hub genes (Figure 7C). A brief overview of the 16 hub genes, including *G6PD*, *ACACA*, *TPI1*, *ALDH7A1*, *GLUD1*, *DLAT*, *GLUL*, *LDHA*, *GLS*, *SHMT2*, *ACLY*, *SHMT1*, *PFKM*, *SUCLA2*, *DLD*, and *SCO2*, is provided. Among these 16 hub genes, eight have been previously reported to be strongly associated with obesity (Matsumura et al., 2024; Khatiwada et al., 2021; Chen et al., 2021; Pan et al., 2023), namely, *G6PD*, *ACACA*, *TPI1*, *DLAT*, *ACLY*, *DLD*, and *ACO2*, highlighting their relevance to metabolic processes.

### 3.7 Construction of the lncRNA‒protein coexpression network

**FIGURE 8:**
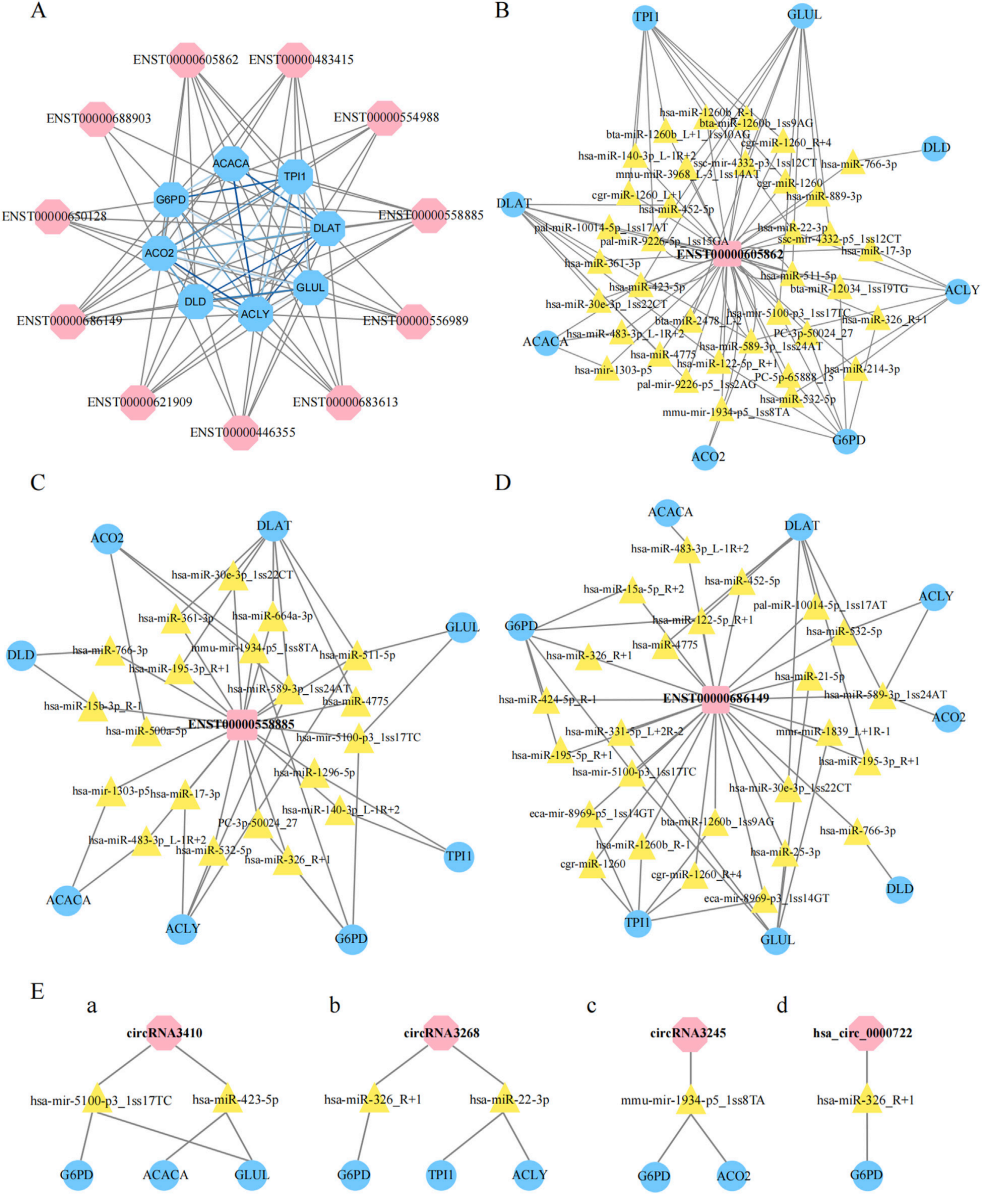
Construction of non-codings co-expression network. **(A)**: Construction of lncRNAs-proteins co-expression network, the blue hexagon represented gene, and the pink hexagon represented lncRNA, the grizzly lines link gene to lncRNA, blue lines link gene to gene. **(B–D)**: Construction of LncRNAs-micRNNAs -proteins co-expression network, the pink square represented lncRNA, the yellow triangle represented micRNA, and the blue oval represented gene. **(E)**: The pink hexagon represented lncRNA, the yellow triangle represented micRNA, and the blue oval represented gene.

We then evaluated the coexpression network of the DE lncRNAs validated by qRT‒PCR and the eight metabolism-related genes highlighted in Figure 7C. In this coexpression network, ENST00000605862, ENST00000558885, and ENST00000686419 were most closely associated with these metabolic genes (Figure 8A). To further investigate the potential regulatory roles of the DE lncRNAs in metabolic pathways, we conducted additional analyses of ENST00000605862, ENST00000558885, and ENST00000686149. ENST00000605862 was targeted by 36 miRNAs, which in turn regulated the eight DE genes (Figure 8B). ENST00000558885 was targeted by 20 miRNAs, and these miRNAs also regulated the eight DE genes (Figure 8C). ENST00000686149 was targeted by 24 miRNAs, with these miRNAs regulating the same eight DE genes (Figure 8D).

In addition, we focused on four differential circRNAs, namely, hsa_circ_0000722, circRNA3410, circRNA3245 and circRNA3268, for coexpression network analysis. We found that circRNA3410 was targeted by two miRNAs, which regulated *G6PD*, *ACACA*, and *GLUL* (Figure 8E/a). CircRNA3268 was also targeted by two miRNAs, which regulated *G6PD*, *TPI1*, and *ACLY* (Figure 8E/b). CircRNA3245 was targeted by mmu-miR-1934-5p_1ss8TA, which in turn targeted *G6PD* and *ACO2* (Figure 8E/c). Finally, hsa_circ_0000722 was targeted by hsa-miR-326_R+1, which specifically targeted *G6PD* (Figure 8E/d).

## 4 Discussion

In this study, we delineated the ncRNA profiles of human omentum adipose tissue in obese and healthy subjects through high-throughput RNA sequencing coupled with comprehensive analysis via multiple databases. Greater omentum adipose tissue, a distinct form of visceral fat, is known to play pivotal roles in the development of obesity. Here, we present a detailed characterization of the involved ncRNAs, with a particular emphasis on the differential expression patterns of lncRNAs, circRNAs, and miRNAs. The ncRNA profiles established in this study are poised to broaden our understanding of the mechanisms underlying obesity, contributing to the growing body of research in this field.

In recent years, with the development of basic research on obesity and related metabolic diseases, lncRNAs have been identified as having substantial potential as biomarkers in fat metabolism-related diseases. Many lncRNAs are adipose-enriched, strongly induced during adipogenesis, and bound at their promoters by key transcription factors, such as peroxisome proliferator-activated receptor γ (PPARγ) and CCATT/enhancer-binding protein α (CEBPα) (Sun et al., 2013). Recent studies have shown that lncRNAs have clinical applicability, are convenient biomarkers for disease diagnosis (An et al., 2020), and, compared with mRNAs, exhibit greater tissue specificity and often function in a tissue-specific manner (Ruan et al., 2020). Studies have shown that lnc-dPrdm16 is required for brown adipocyte differentiation and revealed that conserved lncRNAs are regulators of brown adipogenesis (Ding et al., 2018). To understand more about the biological effects of lncRNAs in WAT, we performed full-gene sequencing of 7 human samples (3 from obese patients and 4 from healthy controls) via RNA sequencing. We ultimately obtained 2642 DE lncRNAs, which with 963 upregulated lncRNAs and 1679 downregulated lncRNAs. We then validated the top 10 DE lncRNAs via qRT‒PCR. The results confirmed that four of the 10 upregulated and seven of the 10 downregulated lncRNAs were consistent with the RNA-seq findings. These lncRNAs represent key targets that merit further investigation.

We then further analyzed these 11 lncRNAs, and the analysis generated multiple findings suggesting that ENST00000605862, ENST00000558885 and ENST00000686149 may play important roles in obesity. Most importantly, we found that the strongest associations were between ENST00000605862 and metabolism-related genes. However, since most human lncRNAs are human- or primate specific, identifying the potential functionality of lncRNAs and further investigating their functions *in vivo* is extremely challenging. Fortunately, a recently published article reported that the human-derived lncRNA ENST00000605862 is homologous to murine-derived ENSMUSG00000092090. These findings may provide a basis for further investigations of the functions of this lncRNA.

Cardiovascular disease (CVD), diabetes, hypertension, and atherosclerosis are associated with the dysregulation of circRNAs in closed cells. Studies *in vitro* and *in vivo* suggest that circRNAs play a role in adipogenesis, WAT browning, obesity, obesity-induced inflammation and insulin resistance. In experiments involving animal adipose tissue, the analysis of pig subcutaneous adipose tissue revealed that circRNA-11897 and circRNA-26852 are DE in obesity (Li et al., 2018). Similarly, Zhang Z. et al. (2019) and Zhang T. et al. (2019) validated the findings in mouse adipose tissue and reported that circARF3 and circNrxn2 are associated with adipose tissue inflammation and WAT browning. Over the past few years, experiments using human adipose tissue have shown that circ-0136134, hsa-circ-0017650, hsa-circRNA9227, circTshz2-1 and circArhgap5-2 are closely related to adipogenesis (Arcinas et al., 2019; Sun et al., 2019). In the present study, we identified 154 DE circRNAs via greater omentum adipose tissue sequencing, with 94 upregulated circRNAs and 60 downregulated circRNAs. We checked the sequencing data and verified the top 10 DE circRNAs. Consistent changes were detected in hsa_circ_0000722, circRNA3410, circRNA3245 and circRNA3268, indicating the high reliability of the sequencing data. These data provide additional potential circRNAs for the regulation of obesity occurrence.

Numerous miRNAs are present in human adipose tissue and influence adipocyte differentiation, β-cell quantity, and the insulin signature (Ghafouri-Fard and Taheri, 2021). Wang et al. (2010) evaluated the expression signatures of 799 miRNAs in human adipocytes during differentiation and in subcutaneous fat samples from nonobese and obese subjects. They identified 50 DE miRNAs between obese and nonobese individuals. In the present study, we utilized human greater omentum adipose tissue and annotated 763 miRNAs, and the results were highly consistent with those in previous reports. Among these miRNAs, 195 were DE, with 94 significantly upregulated mRNAs and 85 significantly downregulated mRNAs. Our genome-wide miRNA profiling study identified additional DE miRNAs, and individual differences may be mainly responsible for these differences. Further research is needed to understand precisely how miRNA activity is involved in the processes underlying the development of obesity,at as well as how miRNAs can be utilized for disease management.

A large number of studies have now reported that targeting ncRNAs is an attractive approach for treating various diseases. However, no LncRNA targeting therapeutics have yet reached clinical translation, and most of the research has been on non-coding and tumor therapy with little research on obesity (Nappi, 2024). Our results highlighted specific lncRNAs ENST00000605862, ENST00000558885, and ENST00000686149 as potential biomarkers for obesity and analyze their functions and roles in metabolic pathways and their relationships with metabolic genes. It was emphasized that lncRNAs may be potential targets as obesity treatment. However, the role of lncRNAs in metabolic pathways lacks experimental validation beyond bioinformatics predictions. The conclusions drawn about the therapeutic potential of the identified ncRNAs are speculative and should be tempered until further experimental validation is performed.

## 5 Conclusion

In summary, our study delineated the ncRNA profiles in omentum adipose tissue from obese human patients. We identified numerous lncRNAs, circRNAs, and miRNAs that are significantly DE. Further ceRNA network analysis of the identified DE ceRNAs with mRNAs revealed that ceRNAs play a significant regulatory role in metabolic processes. Even though integrated analysis across multiple databases identified the lncRNAs ENST000000558885, ENST000000605862, and ENST000000686149 as potential biomarkers for obesity, our study cohort was small and relied on bioinformatic analyses without further experimental validation *in vivo* in animals, so followup should be required to design further experiments to validate the analysis. Overall, our study significantly contributes to the understanding of ncRNAs in obesity.

## Data Availability

The data presented in the study are deposited in the GEO repository, accession number GSE286454.
